# HAT: Hypergeometric Analysis of Tiling-arrays with application to promoter-GeneChip data

**DOI:** 10.1186/1471-2105-11-275

**Published:** 2010-05-21

**Authors:** Erdogan Taskesen, Renee Beekman, Jeroen de Ridder, Bas J Wouters, Justine K Peeters, Ivo P Touw, Marcel JT Reinders, Ruud Delwel

**Affiliations:** 1 Department of Hematology, Erasmus University Medical Center, Rotterdam, 3015 GE, the Netherlands; 2 Delft Bioinformatics Lab (DBL), Delft University of Technology, Delft, 2628 CD, the Netherlands; 3 Netherlands Bioinformatics Centre (NBIC), the Netherlands; 4 Bioinformatics and Statistics, Department Molecular Biology, Netherlands Cancer Institute, Amsterdam, the Netherlands

## Abstract

**Background:**

Tiling-arrays are applicable to multiple types of biological research questions. Due to its advantages (high sensitivity, resolution, unbiased), the technology is often employed in genome-wide investigations. A major challenge in the analysis of tiling-array data is to define regions-of-interest, i.e., contiguous probes with increased signal intensity (as a result of hybridization of labeled DNA) in a region. Currently, no standard criteria are available to define these regions-of-interest as there is no single probe intensity cut-off level, different regions-of-interest can contain various numbers of probes, and can vary in genomic width. Furthermore, the chromosomal distance between neighboring probes can vary across the genome among different arrays.

**Results:**

We have developed Hypergeometric Analysis of Tiling-arrays (HAT), and first evaluated its performance for tiling-array datasets from a Chromatin Immunoprecipitation study on chip (ChIP-on-chip) for the identification of genome-wide DNA binding profiles of transcription factor Cebpa (used for method comparison). Using this assay, we can refine the detection of regions-of-interest by illustrating that regions detected by HAT are more highly enriched for expected motifs in comparison with an alternative detection method (MAT). Subsequently, data from a retroviral insertional mutagenesis screen were used to examine the performance of HAT among different applications of tiling-array datasets. In both studies, detected regions-of-interest have been validated with (q)PCR.

**Conclusions:**

We demonstrate that HAT has increased specificity for analysis of tiling-array data in comparison with the alternative method, and that it accurately detects regions-of-interest in two different applications of tiling-arrays. HAT has several advantages over previous methods: *i) *as there is no single cut-off level for probe-intensity, HAT can detect regions-of-interest at various thresholds, *ii) *it can detect regions-of-interest of any size, *iii) *it is independent of probe-resolution across the genome, and across tiling-array platforms and *iv) *it employs a single user defined parameter: the significance level. Regions-of-interest are detected by computing the hypergeometric-probability, while controlling the Family Wise Error. Furthermore, the method does not require experimental replicates, common regions-of-interest are indicated, a sequence-of-interest can be examined for every detected region-of-interest, and flanking genes can be reported.

## Background

Tiling-arrays are used for the identification of specific genomic DNA regions that can be enriched using various procedures to study certain molecular biological features. For example, DNA fragments that are bound by a protein of interest, e.g., a transcription factor, can be enriched by using Chromatin Immunoprecipitation (ChIP). When these enriched fragments are hybridized to an array, a genome wide protein binding profile can be obtained that is associated with this particular protein of interest in the cell type that was studied (ChIP-on-chip [1]). Other applications of tiling-arrays [2] are: Methylated-DNA immunoprecipitation (MeDIP-on-chip [3]), transcriptome mapping [4], recognition of hypersensitive sites such as segments of open chromatin that are cleaved more readily by DNaseI (DNase-chip [5]), or identification of copy number variations or breakpoints (Array CGH [6]). The use of tiling-arrays to detect enriched DNA regions has several advantages such as *i) *high sensitivity, which allows the detection of small DNA fragments associating with rare molecules and, *ii) *high probe-resolution, which results in accurate acquisition of unbiased data.

A tiling-array is an array of short DNA fragments, which represent 'probes' that cover the entire genome, or contigs of the genome. The hybridization of labeled DNA to an array (for example DNA enriched using ChIP), will produce a quantitative signal intensity for each probe. Multiple contiguous probes with increased signal intensity across a particular genomic region, is a putative region-of-interest, and suggests the presence of a protein binding site.

As there are no standard criteria to accurately define a region-of-interest, a major challenge in the analysis of tiling-array data is to define such a region, and discriminate a positive signal from non-specific signals [7]. Defining regions-of-interest requires intensity thresholds on continuous probe intensity levels. Following this, the decision of the number of consecutive probes above the threshold needs to be made before a region-of-interest is called. This threshold, and the number of probes above the threshold, directly influence the size of the region-of-interest that can be detected. As biologically relevant regions may vary in intensity, employing a single threshold is insufficient. Additionally, as the probe-resolution varies across the genome, and across different tiling-array platforms, choosing a fixed number of consecutive probes as a region-of-interest is also inadequate. Various methods have been developed to detect regions-of-interest in ChIP-on-chip data such as Welch t-test, HMM, TileMap, MAT, Mixture model approach, CMARRT, Starr and Ringo [8-15]. MAT (Model-based analysis of tiling-arrays for ChIP-chip) [11] is one of the most cited methods for analyzing ChIP-on-chip data and it has been shown to outperform Welch t-test, HMM and TileMap [8-10]. MAT uses various user-defined parameters to model a region-of-interest, such as maximum bandwidth, maximum gap size between probes, the minimum number of probes in a region and the use of a fixed threshold. A major limitation of this method is that it assumes a uniform probe-resolution across the genome, and depends on many user-defined parameters.

Here, we propose a statistical framework (HAT: Hypergeometric Analysis of Tiling-arrays) to identify regions-of-interest in tiling-array data. HAT has several advantages over previous methods including MAT: *i) *as there is no single cut-off level for probe-intensity, HAT can detect regions-of-interest for a large number of thresholds, *ii) *it can detect regions-of-interest of any size, *iii) *it is independent of probe-resolution across the genome and across tiling-array platforms and *iv) *it employs only a single user defined parameter: the significance level. HAT can be seen as a generalization of the transcript discovery approach used in Bertone *et al *[4].

**Figure 1:**
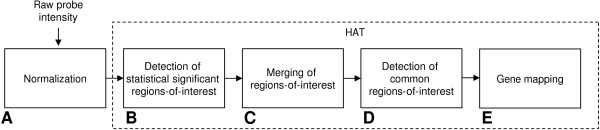
**Illustration of the method**. The different steps of the method, illustrated as blocks (A, B, C, D and E), are needed to process raw probe-intensity data, detection of unique candidate regions and mapping of the detected regions-of-interest to the 5' transcriptional start site of nearby located genes. HAT is indicated with the blocks B, C, D and E. These are representative for the detection of unique candidate regions-of-interest in single, as well as multiple samples.

A detailed description of our framework (Figure 1) can be found in the method section. Briefly, instead of a single probe-intensity cut-off level, HAT evaluates a large number of thresholds. Each threshold transforms the continuous signal intensity levels into discrete calls for each probe; referred to as positive probes where the probe intensity exceeds the threshold, and negative probes where it does not. In order to define regions-of-interest, all probes within the window of each positive probe are evaluated and the *p*-value is defined based on the ratio of both positive and negative probes using the hypergeometric distribution. To detect regions-of-interest of any size, the width of the window is also varied across all relevant window widths, where a relevant window is defined by the expected fragment size in the experimental procedure (e.g., due to sonication). The resulting regions-of-interest for each setting of the threshold and each window width are combined by taking the union of the significant window positions. The Family Wise Error (FWE) is controlled by employing a Bonferroni correction.

We have used two datasets using promoter tiling-arrays to evaluate HAT. In the first assay, tiling-array data was employed to identify genome-wide DNA binding profiles of the transcription factor Cebpa, in a cell line model. Using these data, we have shown that although HAT detected fewer regions-of-interest than MAT, the detected regions are more highly enriched for CEBP binding motifs, and include known Cebpa target genes. In the second experiment, a retroviral insertional mutagenesis assay, HAT identified novel putative transforming loci that may play a role in tumor development. Two of these loci were subsequently validated using PCR.

HAT can also detect and compare regions-of-interest across multiple samples. Each sample is analyzed independently, but when multiple samples within one experiment are used, detected regions-of-interest at the same genomic location among different samples are combined into 'common regions-of-interest', thereby increasing the confidence. In addition, HAT can incorporate sequence information for the detection of pre-defined sequences (e.g., binding location within or near the region). These are highlighted in the graphical output for every detected region-of-interest and indicated in the output file.

## Results and Discussion

### Data

Two distinct experimental datasets were used in this study: ChIP-on-chip data derived from an inducible Cebpa expressing myeloid cell line model and data obtained from genomic DNA from retrovirus induced murine leukemias. Data were generated using the Affymetrix GeneChip Mouse Promoter 1.0 Array. This chip generates 4.6 million perfect match probes over 28000 mouse promoter regions. Promoter regions cover 6 Kb upstream to 2.5 Kb downstream of 5' transcription start sites. Each probe has a size of 25 nt.

### Detection of regions-of-interest for *cebpa *chromatin immunoprecipitation by applying HAT

To compare different methods and to analyze the promoter array data, we made use of a dataset that was obtained from a ChIP of beta-estradiol induced Cebpa in a myeloid cell line, 32D, followed by promoter array hybridizations. The data were used to examine the validity of detected regions-of-interest in two ways: *i) *at the 'CCAAT' binding level; Cebpa interacts with the nucleotide sequence 'CCAAT' within the promoter regions represented on the chip, therefore CEBP binding motifs are expected to be enriched, and *ii) *at the gene level; examination of the presence of known Cebpa target genes, by taking the genes flanking the detected region-of-interest into account. Furthermore, one selected region-of-interest was validated by Real Time Quantitative PCR (qPCR).

The experimental setup was as follows: clones were derived from a myeloid cell line model (32D), that expresses either beta-estradiol inducible Cebpa-ER (3 clones) or control ER (2 clones). Chromatin immunoprecipitations were carried out using an antibody directed against ER in the beta-estradiol treated cells and the DNA obtained from these cells, after immunoprecipitation, was hybridized to Affymetrix promoter chips.

**Figure 2:**
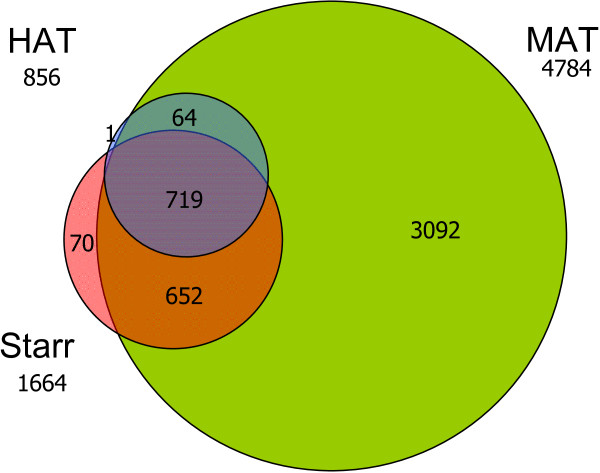
**Venn diagram depiction the overlapping regions-of-interest between HAT, Starr and MAT**. Detected regions-of-interest by HAT (blue: 856), Starr (red: 1664) and MAT (green: 4784) are indicated with the number of overlapping regions between the methods. The overlap of regions detected by all three methods (pink: 719) showed high enrichment for CEBP binding motifs. Overlapping regions between HAT and MAT (64: blue) and Starr and MAT (orange: 652) also showed high enrichment for CEBP binding motifs. Uniquely detected regions by Starr (red: 70) showed no significantly enriched motifs, and MAT (green: 3092) showed limited motifs enriched for CEBP. Note that the number of overlapping regions can contain multiple regions-of-interest detected by a single method.

For method comparison we used Model-based analysis of tiling-arrays for ChIP-chip (MAT), with the default parameters for the detection of regions-of-interest (bandwidth of 300 bp; resulting in 2*bandwidth probe positions, 300 bp of maximum gap size between positive probes, minimum of 8 probes for MAT-score, and enriched fragments at the 1 × 10^-5 ^significance level). The default settings agree with the average sonicated fragment sizes, being 600 bp, and the distance between two consecutive probes being approximately 35 bp. Using the default criteria in MAT, 4784 unique regions-of-interest were detected in at least one of the 32D-Cebpa-ER clones (n = 3) and absent in control samples 32D-ER (n = 2). Using HAT, the same significance level and maximum fragment size (1 × 10^-5 ^and 600 bp respectively) were chosen to detect statistically significant regions-of-interest. Applying these parameters, 1679 statistically significant regions-of-interest were detected in any of the 32D-Cebpa-ER clones; 80% (1318) of these regions were detected in two or more clones (common regions-of-interest). This corresponds to 856 unique chromosomal regions-of-interest. HAT detected approximately one fifth of the regions-of-interest in comparison with MAT for the same significance level, and 99.9% (855) of these unique detected regions in HAT overlapped with the regions detected by MAT (Figure 2).

To investigate the validity of these detected regions-of-interest (for both HAT and MAT) on the sequence level, a motif enrichment analysis was performed. This was carried out using the Cis-regulatory Element Annotation System (CEAS [16]), where a *p*-value is computed for each known motif, and the motifs that are significantly enriched in the regions-of-interest are reported. The top 10 enriched motifs are indicated in Table 1 for both methods. These data showed that HAT detects regions that are highly enriched for the CEBP motif binding sites, whereas MAT does not show a clear enrichment for these sites. Note that the detected regions-of-interest by HAT, are a subset of MAT.

**Table 1:** Motif enrichment analysis.

	MAT				HAT			
**Nr**	**Motif**	**Hits**	**Fold- change**	***p*-value**	**Motif**	**Hits**	**Fold- change**	***p*-value**

1	AP2alpha	9735	1.606	0.0	M00117.CEBPbeta	1532	2.325	2.837E-185
2	Elk-1	5380	1.707	9.226E-286	M00770.CEBP	3076	1.766	2.229E-183
3	M00470.AP-2 gamma	5938	1.641	1.823E-274	M00912.C-EBP	3036	1.715	1.309E-164
4	M00109.CEBPbeta	6170	1.617	3.519E-269	cEBP	1928	1.965	1.886E-157
5	M00695.ETF	3449	1.885	3.049E-250	M00116.CEBPalpha	2689	1.722	4.296E-148
6	M00025.Elk-1	2979	1.949	1.763E-237	M00109.CEBPbeta	1278	2.161	2.349E-132
7	M00446.Spz1	4863	1.665	3.901E-237	M00190.CEBP	2402	1.719	9.670E-132
8	M00008.Sp1	5135	1.625	1.038E-228	M00098.Pax-2	1799	1.578	8.565E-73
9	E74A	3635	1.691	7.076E-188	M00496.STAT1	1909	1.545	8.877E-71
10	M00771.ETS	3756	1.674	2.374E-187	M00971.Ets	1917	1.508	4.418E-64

The top 10 motifs enriched in the detected regions-of-interest (*α *= 1 × 10^-5^) by HAT and MAT for the *cebpa*-study (ChIP-on-chip). Among the top 10 motifs enriched in the regions-of-interest detected with HAT, seven contained the CEBP binding motif whereas for MAT, only one contained the CEBP binding motif. For each reported motif, the number of hits within the regions-of-interest are counted, their fold change computed, and the *p*-value derived using the binomial test.

**Figure 3:**
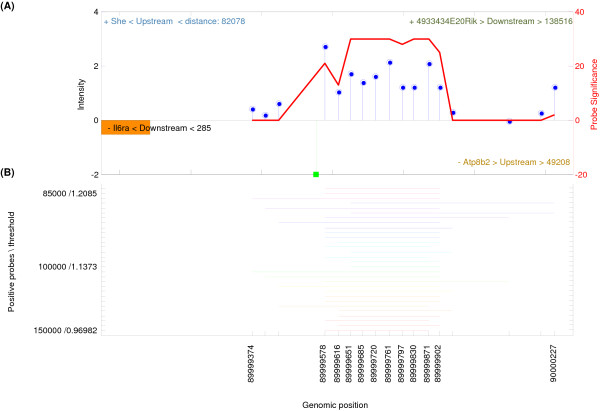
**Graphical output of a detected region-of-interest from the *cebpa*-study**. It was confirmed with qPCR that the Cebpa protein targets and regulates the proximal promoter region of the *il-6 receptor alpha *gene, which lies downstream of the region-of-interest (negative DNA strand). The top panel (A), indicates the probes, represented as vertical blue lollipops, the left y-axis the probe-intensities, and the right y-axis illustrates the contribution of each probe separately to the region (probe-significance). The x-axis indicates the genomic probe positions, and illustrates with a downwards facing green bar; the sequence-of-interest. The sequence, 'CCAAT', was found on the negative DNA strand. Furthermore, flanking genes to this detected region are indicated with distances in base pairs to the 5' transcriptional start site. In the bottom panel (B), the detected regions-of-interest for various windows and probes are shown. The colors represent the detection of regions-of-interest, for a number of different top probes and window sizes. The merged region-of-interest has a fragment width of 853 bp, and lies in the proximal promoter region of *il6ra *on the negative DNA strand.

To investigate detected regions-of-interest based on their flanking genes, regions-of-interest were mapped to the closest 5' transcriptional start site of a gene. Mapping is applied on the forward and reverse DNA strands, with a maximum distance of 300 kb up- and down-stream (NCBI murine genome build 36). This resulted in 2174 unique genes for the 856 unique detected regions-of-interest using HAT (10.7% out of the total set of unique genes present in mouse). These mouse genes were subsequently overlayed with 169 known homologous human Cebpa target genes (derived from Ingenuity Pathway Analysis, IPA), demonstrating that 40 Cebpa target genes being detected by HAT (*p *≤ 4 × 10^-7^) and 86 by MAT (*p *≤ 3 × 10^-5^). Note that MAT has detected approximately five times more regions-of-interest (4784) resulting in 7238 unique genes (35.8% out of the total set of unique genes present in mouse). Some of the detected Cebpa target genes have previously been described, such as: *myc*, *hp*, *mpo *and *il6ra *[17-20]. Enrichment of the *il-6 receptor alpha *(*il6ra*) transcriptional start site (Figure 3) was subsequently validated by qPCR.

An alternative comparison can be performed using the number of regions-of-interest, instead of the significance level. For HAT; 856 unique regions-of-interest were detected with a significance level *α *= 1 × 10^-5^. To gain approximately the same number of regions-of-interest using MAT, we would need to set the *α *level at 1 × 10^-19^, resulting in 893 regions-of-interest. The regions-of-interest detected by HAT showed 84% (718/856) overlap with MAT whereas the overlap of detected regions of MAT with HAT was 83% (742/893). Both methods show a high enrichment for the CEBP binding motifs. Comparing the detected regions-of-interest with respect to MAT (4827 for *α *= 1 × 10^-5^), we need to set the *α *level higher than 0.05 in HAT, but this may compromise the reliability of detected regions-of-interest. For this reason, we have set the *α *level at 0.05 and hereby detected 1910 unique regions-of-interest. These were highly enriched for CEBP binding motifs based on the motif enrichment analysis (Table 2), whereas the detected regions-of-interest by MAT were not highly enriched for CEBP binding motifs (Table 1). The regions-of-interest detected by HAT showed 98% (1879/1910) overlap with MAT whereas the overlap of detected regions of MAT with HAT was 39% (1874/4784).

**Table 2:** HAT: Motif enrichment analysis using *α *= 0.05.

Nr	Motif	Hits	Fold Change	*p*-value
1	M00117.CEBPbeta	3236	2.082	6.187E-304
2	M00770.CEBP	6688	1.628	8.947E-299
3	M00912.C-EBP	6609	1.583	6.697E-265
4	M00116.CEBPalpha	5858	1.591	1.121E-239
5	cEBP	4068	1.758	1.875E-238
6	M00190.CEBP	5245	1.592	2.814E-215
7	M00716.ZF5	3927	1.706	2.121E-208
8	M00109.CEBPbeta	2645	1.896	3.059E-195
9	M00098.Pax-2	4355	1.619	1.761E-191
10	M00428.E2F-1	4374	1.572	4.665E-171

The top 10 motifs enriched in the 1910 detected regions-of-interest using HAT (*α *= 0.05) in the *cebpa*-study. There is a high enrichment for binding motif CEBP. For each reported motif, the number of hits within the regions-of-interest are counted, their fold change computed, and the *p*-value derived using the binomial test.

In addition, the HAT and MAT results were also compared with the detected regions of Starr [14]. Starr implements the CMARRT algorithm [13] and thereby incorporates the correlation structure for the identification of regions-of-interest in tiling-array data. For the detection of regions-of-interest, we have utilized similar parameter settings (fragment size = 600 bp, minimum number of probes in a region = 8 and *α *= 1 × 10^5^) as used in HAT and MAT. Using these parameter settings, Starr detected 1664 regions-of-interest and showed high enrichment for CEBP binding motifs (Additional file 1: Supplemental Table S1). Following this, we have examined the overlap of regions-of-interest detected by all methods as depicted in Figure 2. All regions-of-interest detected by HAT (except one) were also detected by MAT alone or together with Starr (64 and 791 respectively). Note that the number of overlapping regions can contain multiple regions-of-interest detected by a single method. To asses the validity of the detected regions-of-interest by HAT, Starr and MAT, we have examined the enrichment for CEBP binding motifs for the different parts in the Venn diagram, depicted as different colors in Figure 2 (blue, red, green, orange and pink). High enrichment for CEBP motifs are found for; *i) *the overlap of HAT with the other two methods (pink: 719), *ii) *the overlap of HAT with MAT (blue: 64) and, *iii) *the overlap between Starr and MAT (orange: 652). No significant enriched motifs are found in the regions detected only by Starr (red: 70) and limited motifs are enriched for CEBP in the regions detected only by MAT (green: 3092). Therefore we can conclude that HAT had the highest specificity as it was able to detect regions-of-interest highly enriched for CEBP binding motifs.

### Detection of retroviral insertion sites by HAT

Retroviral Integration Mutagenesis (RIM) in mice is a powerful tool to identify new genes playing an important role in oncogenesis. Mice are injected with retroviruses that potentially integrate into the murine genome upon infection. Viral integration can lead to gene deregulation, and depending on the genes affected, tumors may develop. Genes located proximal to viral integration sites are potentially oncogenic, leading to tumor development. Genomic regions that have been targeted by proviral DNA in multiple tumors are called common viral integration sites (VIS), and are likely driving tumor development. Using retroviral insertional mutagenesis, many oncogenes have been identified using large sequencing screens in multiple tumors [21-24]. We hypothesise that within tumors, genes may be silenced as a result of proviral integration caused by hypermethylation of the CpGs in the viral long terminal repeat, and subsequently in the promoters of their target genes. The identification of methylated genes by means of retroviral insertional mutagenesis may be studied by Methyl-DNA immunopreciptitation (MeDIP-on-chip), followed by inverse PCR, using long terminal repeat (LTR) specific primers. After combining these two technologies, we hybridized samples to Affymetrix promoter chips to identify genomic locations involved in viral integration that potentially harbour new tumor suppressor genes (TSG).

**Figure 4:**
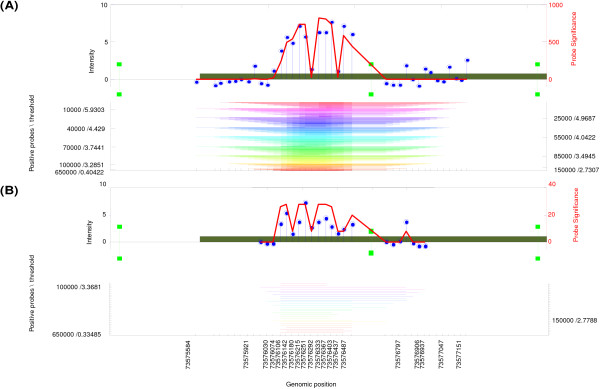
**Graphical output of a detected cmVIS in the MeDIP-study**. A region-of-interest detected in two samples, is illustrated in Panels A and B. Panel A shows 840 sub-regions that are merged with a total length of 1567 bp. The restriction sites, indicated as green bars, are located in and around the detected region, and are present on both DNA strands due to the palindrome sequence: 'GATC'. The region-of-interest detected in the second tumor (Panel B), exists of 28 subregions, with a fragment width of 949 bp.

Regions-of-interest within this dataset differ from the *cebpa*-study as they have; *i) *a higher variability in fragment sizes and, *ii) *contain specific sequences within the identified regions. Therefore these data are used to examine the performance and broad applicability of HAT among different applications of tiling-array data. Using HAT, we have identified candidate TSGs in mouse tumors by considering regions with a maximum fragment size of 1000 bp and a significance level *α *= 0.05. With these parameters, we detected 15 methylated Viral Integration Sites (mVIS); of which one appeared to be a common methylated VIS (cmVIS) among two samples (Figure 4).

**Figure 5:**
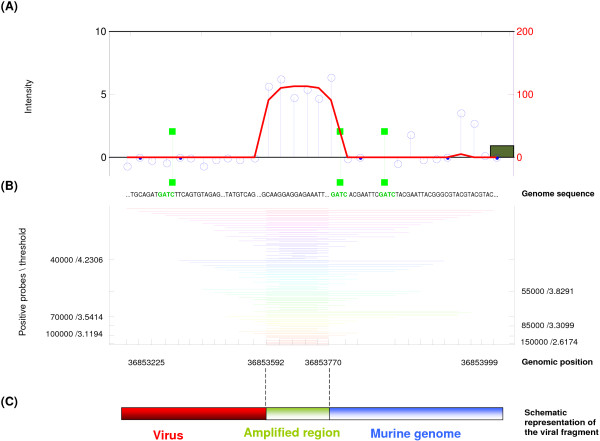
**Graphical output of a detected and validated mVIS in the MeDIP-study**. Panel A illustrates the detected mVIS which are subject to DNA methylation. Only a section of the detected region-of-interest has an increased probe-intensity; the probe-significance signifying this subregion. Directly beside the increased probe-significance, a restriction cleavage site is indicated by means of a green bar. Due to the palindrome sequence, these sites are indicated at the same genomic position on both DNA strands. Panel B shows the detected statistically significant regions among the different thresholds, and window sizes with various colors. A schematic representation of the amplified genomic region, with the virus- and the murine contribution, is shown in Panel C.

Besides the detection of candidate regions based on a statistical framework, we have attached additional mouse genomic sequence information (MM8) to the model, in order to determine the sequence-of-interest based on the restriction enzyme used in the inverse PCR. Within this assay, a restriction enzyme (DpnII) will cleave DNA at sequence 'GATC', within the integrated viral sequence and the flanking genome. Note that because of this property, it is expected that every detected region must contain a nearby restriction site, which can easily be verified with HAT. HAT showed that all detected mVISs contain a nearby restriction site, conforming specificity of the identified region as being a viral insertion site. For PCR validation of the method, two mVISs were selected based on their location to a nearby 5' transcriptional start site, and confirmed. One of the validated regions is illustrated in Figure 5.

### Extended applications of HAT

The scope of this method is not limited to the presented studies (i.e., detecting transcription factor binding sites and DNA methylated regions). Moreover, we have successfully applied HAT for the detection of regions enriched for histone modifications such as, trimethylation of histone 3 at lysine 4 or lysine 27 (H3K4 me3 and H3K27 me3) (data not shown). Some of the detected regions-of-interest were selected for further validation and confirmed by qPCR. Regarding tiling-array data spanning the entire genome [25] (e.g., RNA transcript mapping data [4]), we do not expect changes in algorithm performance (detection of regions-of-interest) due to an increased variability in hybridization consistency because the applied normalization method [11,26] corrects for two major causes of differences in hybridization consistency, i.e., probe sequence and presence of repeats within the genome. Furthermore, in addition to one-color arrays (e.g., Affymetrix tiling-arrays) we envision that HAT can also be applied on data stemming from two-color arrays (e.g., Nimblegen tiling-arrays), because data structure remains similar. We stress however that the normalization procedure is an important step and strongly depends on the type of tiling-array dataset.

## Conclusions

Here we propose a statistical framework; HAT (Hypergeometric Analysis of Tiling-arrays) to analyze tiling-array data. We showed that the method is robust and has increased specificity in the detection of regions-of-interest in comparison with two alternative methods. This is achieved by computing the hypergeometric-probability for every detected region-of-interest, among different threshold levels of probe-intensities and window sizes, while keeping control of the Family Wise Error (FWE) by employing Bonferroni correction. Besides the detection of regions-of-interest, HAT also determines sequences-of-interest, flanking genes and the distances to 5' transcriptional start sites on both DNA strands. We describe the performance of HAT, when applied to different experimental tiling-array datasets. For each experimental dataset, the selected downstream genes flanking the detected regions-of-interest were successfully confirmed by (q)PCR. We compared the detected regions-of-interest of HAT with two other methods (MAT [11] and Starr [14]), and showed that HAT resulted in a reduced number of detected regions-of-interest using the same significance for both MAT and Starr. However, using motif enrichment analysis we showed that the regions-of-interest detected by HAT were more enriched for the expected binding motifs of CEBP compared to MAT and showed similar enrichment for Starr, illustrating increased specificity using HAT.

Besides analyzing ChIP-on-chip data, HAT is also suitable for the analysis of other types of tiling-array data. Applying HAT to the data from the MeDIP inverse-PCR and promoter-GeneChip hybridization experiment, we discovered mVISs and cmVIS that are subject to DNA methylation and identified the genes (unpublished data) that flank these methylated viral integration sites (Figure 4 and 5).

HAT is applicable to detect regions-of-interest among the different applications of tiling-arrays, and has the advantage of being independent for thresholds, number of probes in a region and probe-resolution. It does not depend on setting various user defined parameters, except for the significance level and an optional maximum fragment size.

## Methods

Extracting candidate gene-regions based on high throughput data using tiling-arrays is a multi-step process (Figure 1). The first step is to normalize the probe-intensity data from the chip (Figure 1A). For this purpose, we utilize the normalization from Model-based analysis of tiling-arrays for ChIP-chip (MAT) [11,26], but other normalization procedures can also be applied. The normalization procedure prevents systematic variation between experimental conditions, which are unrelated to biological difierences. As a result of this normalization, the probe-intensity values follow a normal distribution with a negative mean; hence the majority of probes have values below zero, and are ignored in all subsequent analyses. Probe-intensities that may be the result of hybridization of labeled DNA on the chip (e.g., were present in the immunopreciptitated chromatin sample), have values greater then zero and are used to determine candidate regions-of-interest.

**Figure 6:**
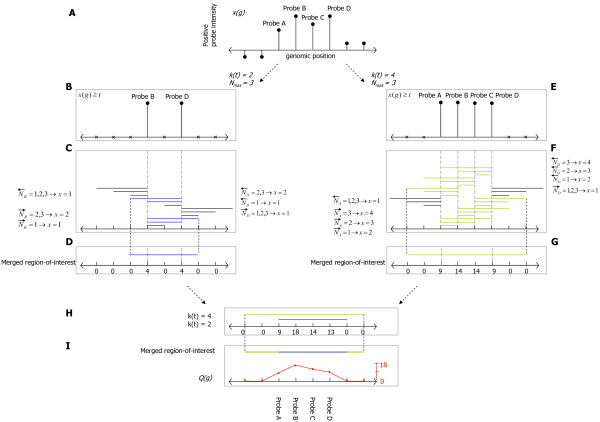
**Schematic depiction for the detection of regions-of-interest**. Schematic depiction for the detection of regions-of-interest, based on probe-intensities. Eight probes, with their genomic location, are shown in Panel A. Four of these have positive probe-intensities. The use of multiple thresholds, transforms continuous data into discrete data; as shown in Panel B and E. Various window scales *N*, are used to examine neighboring probes for their probe-intensities in Panel C and F. These windows will contain different number of positive probes. The hypergeometric probability is computed for every region-of-interest, and excludes a region-of-interest when the region is not statistically significant after correcting for a single positive probe in a region-of-interest and multiple testing. The remaining regions are merged for each *k(t) *(illustrated in Panel D, G, H) and then among all *k(t) *to a single region-of-interest (Panel I). To determine how often probes were detected in statistically significant regions, the probe-significance is computed (Panel D and E), and indicated with a red colored line that signifies the statistically significant probes in the detected region-of-interest.

After normalization, probe-intensities are discretized using a varying threshold and the significance of the probes within a varying window is determined. Significant window positions are then merged into the final regions-of-interest. We illustrate this approach in the simplified schematic representation shown in Figure 6. In Figure 6A, eight probes are shown at an arbitrary genomic location. Their intensities are represented by vertical lollipops. The positive probes (six in this example) are assumed to be part of a possible candidate region. Probes with higher intensity levels are more likely to be the results of hybridization on chip, but the exact level of intensity for which this is the case is unknown. Therefore, multiple probe intensity levels are taken into account by varying the discretization threshold *t*. The number of probes that exceed this threshold (called positive probes) is denoted by *k*(*t*). Figure 6B and 6E, illustrates the thresholds *k*(*t*) = 2 and *k*(*t*) = 4, respectively. All probes exceeding *t *are set to one, and those not exceeding the threshold *t *are set to zero.

To define a region-of-interest, we determine the significance of all possible window positions *g*, for which the window contains at least one positive probe. To account for the fact that the exact number of probes in a region-of-interest is undefined, and may differ greatly between different regions-of-interest due to differences in local probe-resolution; the window width *N *is varied. To prevent evaluating many highly similar windows, thereby incurring a high multiple testing penalty, only those window widths for which the number of probes in the window varies are evaluated. Therefore, *N *is defined in terms of the number of probes contained in the window. The number of positive probes in a window of width *N*, at genomic position *g*, for threshold value *t*, is denoted by *x*(*g*, *t*, *N*). In the example presented in Figure 6, we varied *N *from 1 through to 3. For the case *k*(*t*) = 2 (Panel B and C), *x*(*g*, *t*, *N*) ranges from 1 through to 2, and in case *k*(*t*) = 4 (Panel E and F), *x*(*g*, *t*, *N*) ranges from 1 through to 4.

For each window, a *p*-value is determined; defined as the probability of observing at least *x *positive probes in the window. For any window position *g*, threshold level *t *and window width *N*, *p*(*g*, *t*, *N*) is computed as:(1)

Note; that since we restrict each window to contain at least one positive probe to prevent evaluating useless window positions, this probability is conditioned on *X *≥ 1. All probabilities are computed using the hypergeometric distribution:(2)

where *K *is a fixed parameter and represents the total number of probes present on the (e.g., promoter) chip. To correct for the number of tests performed, we apply Bonferroni correction, controlling the Family Wise Error per value of the threshold level as follows:(3)

Based on this p-value, it is possible to exclude regions that do not reach a predefined significance level (*α*):(4)

Due to the use of various values for *t *and *N*, similar or partly overlapping regions are found. In order to find a single region-of-interest at the same genomic location, these overlapping regions are merged by joining regions with one or more overlapping probes. In our example, we assume for simplicity, that windows with *x*(*g*, *t*, *N) *≥ 2 are statistically significant. These statistically significant regions are colored blue and green in Figure 6C and Figure 6F respectively. The merging procedure is illustrated in Figure 6D, where four blue regions are merged into a single region, and in Figure 6G where 18 green regions are merged.

Finally, regions found for different threshold levels *t *are also merged (Figure 6H) into the final region-of-interest (Figure 6I). Regions-of-interest tend to be larger than the regions detected at a single setting of the threshold level, or single window width due to the merging of all these individual regions. To determine the most important parts of the region-of-interest, we introduce a probe-significance score *Q*(*g*), which reports how often probes were part of the statistically significant region. This score is illustrated by the red curve in Figure 6I, and computed as follows:(5)

In our example so far, regions are detected within a single sample. When multiple samples are available (for the same experiment), array-wise detection of regions-of-interest is examined in order to detect common regions-of-interest (Figure 1D). A radius, defined in base pairs, can be defined to set the maximum distance between regions over multiple samples (default is zero).

### Additional properties of HAT

The HAT method includes two additional properties beside the detection of regions-of-interest; *i) *The determination of sequences-of-interest surrounding and within the detected regions-of-interest, e.g., the enhancer binding protein Cebpa is known to interact with 'CCAAT' sequences, and it is therefore expected that detected regions-of-interest contain this sequence in a chromatin IP experiment. The presence, and positions of the sequences-of-interest can be indicated in the (graphical) output of HAT. In this graphical output, sequences are indicated with an upward facing green bar, indicating that the sequence is detected on the positive strand, or a downward facing green bar representing a sequence on the negative strand. *ii) *The determination of genes flanking the detected regions-of-interest. For every detected region-of-interest (for both up- and down-stream and forward and reverse DNA strands), the genes with the closest distance to the transcriptional start site are determined, and indicated in the (graphical) output.

To include these regions-of-interest and genes into the HAT method, the public genome-sequence (available for different model systems) can be utilized from the UCSC genome browser.

## Availability and requirements

HAT is implemented in Matlab R2009b and is tested on Unix and MS-Windows. It is available on http://www.erasmusmc.nl/hematologie/. The run time depends on the number of used threshold cut-off's as the computation complexity increases linear with the used number of probes for the detection of regions-of-interest. In addition, run time also depends on the different steps in the method (Figure 1B-F). On average, for the *cebpa*-study, 28 minutes were needed per sample for the detection of regions-of-interest, while MAT required on average a run time of 23 minutes per sample. Note, however, that in our algorithm the data were analyzed using a multitude of window sizes and thresholds. A more detailed overview of the run time for each step in the method can be found in Additional file 2: Supplemental Figure S1.

## Authors' contributions

ET, JdR, and MJTR contributed to the conceptual design of the study. ET performed the analysis. RB and BJW performed the biological experiments whereas RB and RD provided biological insights. MJTR, RD, RB, JKP and IPT participated in the discussion of the results. ET, JdR, JKP and RB wrote the manuscript. All authors read and approved the final manuscript.

## Supplementary Material

Additional file 1 **Table S1 - Starr: Motif enrichment analysis**. The top 10 motifs enriched in the 1664 detected regions-of-interest using Starr (fragment size = 600 bp, minimum number of probes in a region = 8, *α *= 1 × 10^-5^) in the *cebpa*-study. There is a high enrichment for binding motif CEBP. For each reported motif, the number of hits within the regions-of-interest are counted, their fold change computed, and the *p*-value derived using the binomial test. Click here for file

Additional file 2 **Figure S1 - HAT Computation performance**. Run time of the various steps in the method. The *cebpa*-study is used to analyze the run time for the different steps in the method; Step B: loading data and detection of regions-of-interest, Step C: Merging of regions-of-interest and computation of the probe-significance, Step D: detection of common-regions-of-interest and Step E: gene mapping. Per sample, 62 minutes were needed on average to process all the steps in the method. Click here for file
