# Supplementary material for: HAT: Hypergeometric Analysis of Tiling-arrays with application to promoter-GeneChip data
Source: BMC Bioinformatics. 2010 May 21;11:275. doi: 10.1186/1471-2105-11-275 (PMC2892465; doi:10.1186/1471-2105-11-275)
Supplement: Additional file 1 — Table S1 - Starr: Motif enrichment analysis. The top 10 motifs enriched in the 1664 detected regions-of-interest using Starr (fragment size = 600 bp, minimum number of probes in a region = 8, α = 1 × 10^-5^) in the cebpa-study. There is a high enrichment for binding motif CEBP. For each reported motif, the number of hits within the regions-of-interest are counted, their fold change computed, and the p-value derived using the binomial test. [file 1471-2105-11-275-S1.PDF]

Starr: Motif enrichment analysis

Nr	Motif	Hits	Fold Change	p-value
1	M00117.CEBPbeta	1992	2.153	2.738E-203
2	M00912.C-EBP	4084	1.643	9.462E-190
3	cEBP	2570	1.866	2.944E-181
4	M00770.CEBP	3980	1.627	1.902E-178
5	M00116.CEBPalpha	3523	1.607	7.147E-151
6	M00109.CEBPbeta	1670	2.011	5.445E-145
7	M00190.CEBP	3138	1.599	3.134E-132
8	HLF	666	2.023	3.795E-60
9	M00260.HLF	608	2.017	9.943E-55
10	M00771.ETS	845	1.731	6.804E-49
