# Supplementary figures and images for: HAT: Hypergeometric Analysis of Tiling-arrays with application to promoter-GeneChip data
Source: BMC Bioinformatics. 2010 May 21;11:275. doi: 10.1186/1471-2105-11-275 (PMC2892465; doi:10.1186/1471-2105-11-275)

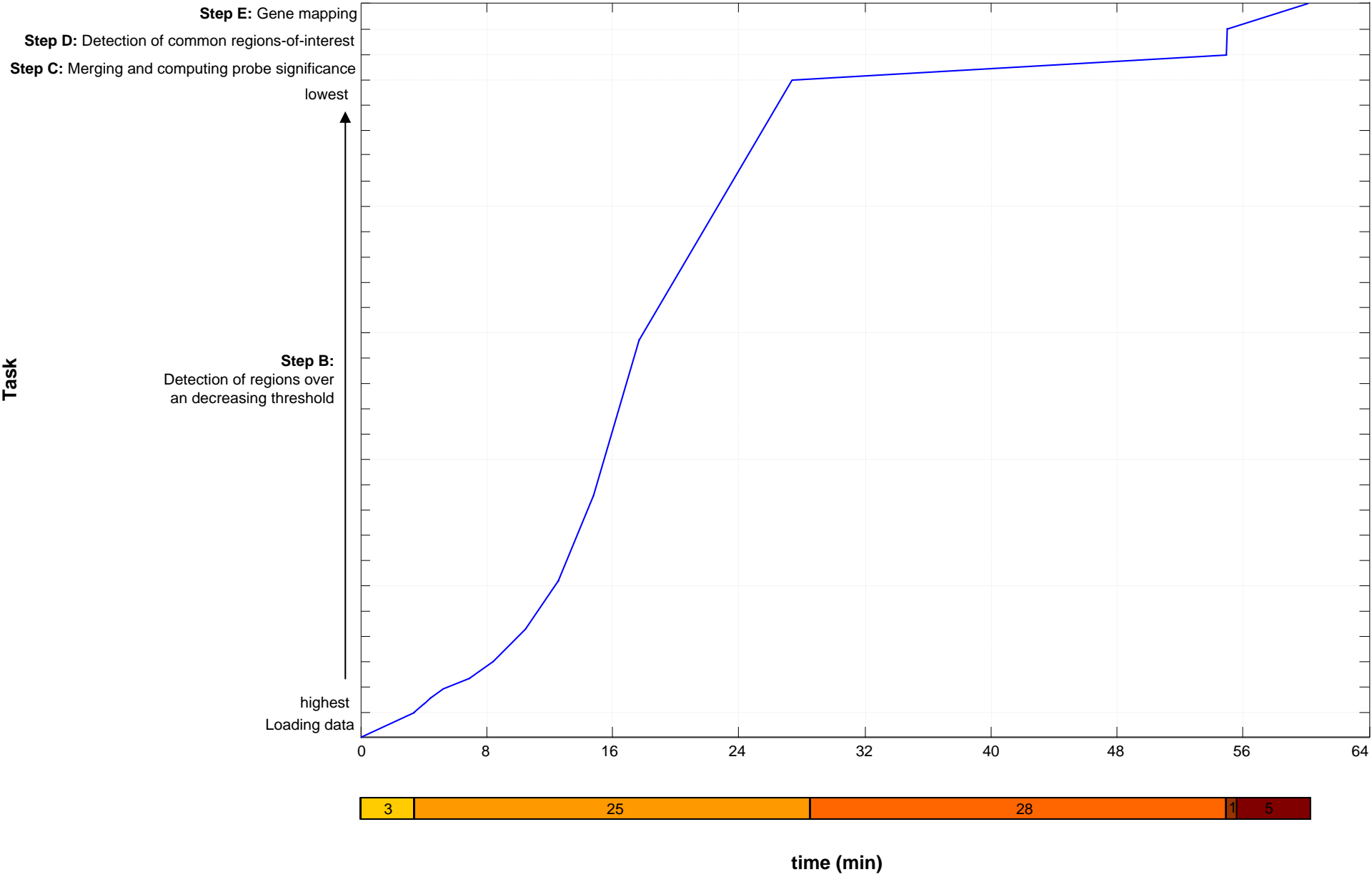

Supplement: Additional file 2 — Figure S1 - HAT Computation performance. Run time of the various steps in the method. The cebpa-study is used to analyze the run time for the different steps in the method; Step B: loading data and detection of regions-of-interest, Step C: Merging of regions-of-interest and computation of the probe-significance, Step D: detection of common-regions-of-interest and Step E: gene mapping. Per sample, 62 minutes were needed on average to process all the steps in the method. [file 1471-2105-11-275-S2.PDF]
